# Effect of Ce-Y Composite Addition on the Inclusion Evolution in T91 Heat-Resistant Steel

**DOI:** 10.3390/ma18071459

**Published:** 2025-03-25

**Authors:** Jun Liu, Gen Li, Chengbin Shi, Zhengxin Tang, Lei Jia, Yu Zhao, Shijun Wang, Xikou He

**Affiliations:** 1 State Key Laboratory of Advanced Metallurgy, University of Science and Technology Beijing, No. 30 Xueyuan Road, Haidian District, Beijing 100083, China; m202221387@xs.ustb.edu.cn (J.L.);; 2 Research Institute of Special Steel, Central Iron and Steel Research Institute Co., Ltd., No. 76 Xueyuan South Road, Haidian District, Beijing 100081, China

**Keywords:** Ce-Y composite addition, T91 steel, inclusions evolution, thermodynamic calculation, rare earth elements

## Abstract

This study investigates the influence of rare earth elements Ce and Y on the evolution of inclusions in T91 steel by melting experimental steels with varying Ce-Y contents in a vacuum induction melting furnace. The results show that the inclusions in the steel without rare earth are mainly composed of Mg-Al-O oxides, (Nb, V, Ti)(C, N) carbonitrides, and composite inclusions formed by carbonitrides coated oxides, and all of them have obvious edges and corners. Upon the addition of different concentrations of Ce and Y, the oxygen content in the steel significantly decreased, and the inclusions were modified into spherical rare earth oxides, sulfides, and oxy-sulfides. Additionally, no large-sized primary carbonitrides were observed. The average size of the inclusions was reduced from 2.8 μm in the non-rare-earth-added steel to 1.7 μm and 1.9 μm with rare earth addition. Thermodynamic analysis indicates that the possible inclusions precipitated in the steel with varying Ce contents include Ce_2_O_3_, Ce_2_O_2_S, Y_2_O_3_, Y_2_S_3_, and CeS. With the increase in Ce content, the rare earth inclusions Y_2_S_3_, Y_2_O_3,_ and CeS can be transformed into Ce_2_O_2_S and Ce_2_O_3_. There are two kinds of reactions in the process of high-temperature homogenization: one is the internal transformation reaction of inclusions, which makes Y easier to aggregate in the inner layer, and the other is the reaction of Y_2_S_3_→CeS and Y_2_O_3_ + Y_2_S_3_→Ce_2_O_2_S due to the diffusion of Ce in the matrix to the inclusions. Combined with the mismatch analysis, it can be seen that Al_2_O_3_ has the best effect on the heterogeneous nucleation of carbonitrides during the solidification of molten steel. Among the rare earth inclusions, only Ce_2_O_3_ may become the nucleation core of carbonitrides, and the rest are more difficult to form heterogeneous nucleation. Therefore, by Ce-Y composite addition, increasing the Y/Ce ratio can reduce the formation of Ce_2_O_3_, which can avoid the precipitation of primary carbonitride and ultimately improve the dispersion strengthening effect. This study is of great significance for understanding the mechanism of rare earth elements in steel and provides theoretical guidance for the composition design and industrial trial production of rare earth steel.

## 1. Introduction

Because of its excellent high-temperature strength and creep resistance, T91 steel has been widely used in the field of new generation high parameter thermal power or fourth-generation nuclear power [1,2]. As martensitic strengthening steel, the increase in Al content in T91 steel has great damage to the rupture strength and rupture plasticity [3]. It is usually required that the Al content does not exceed 0.02 wt.%, and N/Al ≥ 4. On the one hand, the decrease in Al content is helpful to reduce the content of Al_2_O_3_ inclusions in steel and avoid the stress concentration caused by the large thermal deformation ability difference between high-hardness Al_2_O_3_ inclusions and steel matrix, which leads to the formation and propagation of microcracks [4]. On the other hand, low Al content helps to prevent the combination of Al and N during solidification to form AlN, thereby reducing the amount of precipitation of the strengthening phase (Nb, V)(C, N). At the same time, the AlN distributed along the grain boundary will become the nucleation core of creep voids, which promotes the cracking of M_23_C_6_ nucleated on it to form voids, which has an adverse effect on the creep resistance of T91 steel [3]. However, low Al content may lead to the problem of excessive O content in steel, so the addition of rare earth is considered to solve the above problems.

Rare earth has the effect of purifying molten steel [5,6,7], microalloying [8], and modifying inclusions [9,10,11,12,13] in steel, thus improving the microstructure and properties of steel. Especially for martensitic heat-resistant steel, the addition of rare earth Cerium (Ce) or Yttrium (Y) can improve the oxidation resistance of steel [14], high-temperature creep properties [2], and hot ductility [15]. However, its reaction mechanism in molten steel is not clear, and a stable addition process has not yet been formed. After adding molten steel, it is easy to react with [O] and [S] to form spherical rare earth inclusions with high melting points and small sizes [10,12], which affects the yield and performance stability. Jiang et al. [12] added rare earth Ce (0.01, 0.024, 0.042 wt.%) to C110 oil casing steel and found that with the extension of reaction time, the order of inclusion modification was CaO·Al_2_O_3_→CeAlO_3_→Ce_2_O_3_/Ce_2_O_2_S, and the final product was related to the amount of Ce added. In addition, rare earth inclusions may still change during solidification and heat treatment. Ren et al. [16] studied the transformation of inclusions in ultra-low carbon Al-killed steel with 0.0033, 0.0083, and 0.014 wt.% rare earth Ce during solidification and cooling. It was found that during solidification and cooling, homogeneous CeAlO_3_ inclusions were transformed into Al_2_O_3_-Ce_2_S_3_ composite inclusions in steel containing 0.0033 wt.% Ce, and Ce_2_O_2_S inclusions were transformed into CeAlO_3_-Ce_2_S_3_ or Al_2_O_3_-Ce_2_S_3_ composite inclusions in steel containing 0.0083 wt.% Ce. However, the inclusions in the steel containing 0.014 wt.% Ce almost did not change. Zhang et al. [17] added 0.0085 and 0.0180 wt.% Y to 18Cr-8Ni heat-resistant steel and studied the evolution of yttrium-based oxide inclusions during heat treatment at 1473 K. After heating, an oxide phase with high yttrium content was precipitated from the original inclusions, which led to the emergence of Y-rich and Al-rich parts in the steel. In addition, the effect of rare earth on the precipitated phase cannot be ignored. Li et al. [18] found that rare earth has no modification effect on TiN, while Al_2_O_3_ inclusions reduce the nucleation core of TiN by modifying into spherical CeAlO_3_ inclusions, thus reducing the size of composite inclusions. The addition of Ce will greatly reduce the number of TiN and TiN-Al_2_O_3_ composite inclusions with regular geometric polygons in the steel, forming fine spherical Ce-Ti-N inclusions or Ce-Al-O composite inclusions.

Previous studies have demonstrated that the individual addition of rare earth elements, either Ce or Y, can effectively modify inclusions in various types of steel materials, thereby enhancing their mechanical properties. Ce and Y exhibit similar thermodynamic properties; however, due to slight differences in atomic radius and electronegativity, they may demonstrate distinct behaviors in specific applications. Additionally, there is limited research on the evolution behavior of inclusions in molten steel with the combined addition of Ce and Y, the influence of Ce-Y rare earth inclusions on the precipitation phases during solidification, and the impact of heating on the evolution of Ce-Y rare earth inclusions. Therefore, it is necessary to further study the mechanism of two or more rare earth elements on the properties of steel materials. In this study, based on the effect of rare earth on inclusions in T91 steel, the morphology, composition, and distribution characteristics of inclusions in T91 steel without rare earth and Ce-Y composite addition of rare earth were discussed in detail. Through classical thermodynamic analysis, mismatch theory, and inclusion growth kinetics, how rare earth elements act on the formation and growth process of inclusions was revealed, and the influence mechanism of Ce-Y composite addition on the evolution of inclusions was discussed in depth so as to provide theoretical guidance for the industrial production of rare earth steel.

## 2. Materials and Methods

A 50 kg vacuum induction melting furnace (VIM) was used to smelt the test steel. Pure iron, monocrystalline silicon, and pure metal raw materials such as manganese, niobium, vanadium, chromium, and molybdenum were added to the crucible in proportion. After melting into liquid in a high vacuum environment (<10 Pa), Ni-Mg was added to deoxidize, and then graphite was added to avoid a large amount of carbon loss caused by the carbon–oxygen reaction. Then, after filling Ar to 30,000 Pa, ferrochromium nitride was added. For the furnaces that need to add rare earth, pure rare earth Ce and Y are added 5 min before pouring steel, and finally, the ingot was poured. The ingot was homogenized at 1200 °C for 24 h and forged into φ15 mm bars at 1150 °C.

The contents of Ce, Y, Nb, Mn, Mo, V, and Si were determined by inductively coupled plasma atomic emission spectrometry (ICP-AES). The contents of Al and Ti were determined by inductively coupled plasma mass spectrometry (ICP-MS). The content of Cr was measured using the titration method, the content of N was measured using the thermal conductivity method, and the contents of C, O, and S were measured using the infrared absorption method. The specific chemical composition is shown in Table 1. The rare earth content of B0 and C0 steel was 0.015 wt.% Ce + 0.012 wt.% Y and 0.022 wt.% Ce + 0.042 wt.% Y, respectively. The forging bar samples were cut and made into 10 × 10 × 15 mm metallographic samples by wire cutting. After grinding and polishing, the morphology and size of inclusions in steel were observed with a scanning electron microscope (SEM, JSM-IT800, JEOL, Tokyo, Japan). The composition of inclusions was analyzed by energy-dispersive spectroscopy (EDS, Oxford X-Max, Oxford Instruments, Oxford, UK). Finally, the composition, quantity, and size distribution of various inclusions in samples with different rare earth contents were counted using an inclusion automatic scanning analyzer (ASPEX EXPLORER, FEI Corporation, Hillsboro, OR, USA). The detection surface was the longitudinal section of the forging bar, beam energy was 20 kV, Working Distance was 17.438 mm, and the scanning area was 61.44 mm^2^.

## 3. Results and Discussion

### 3.1. Morphology and Distribution of Inclusions

**Figure 1:**
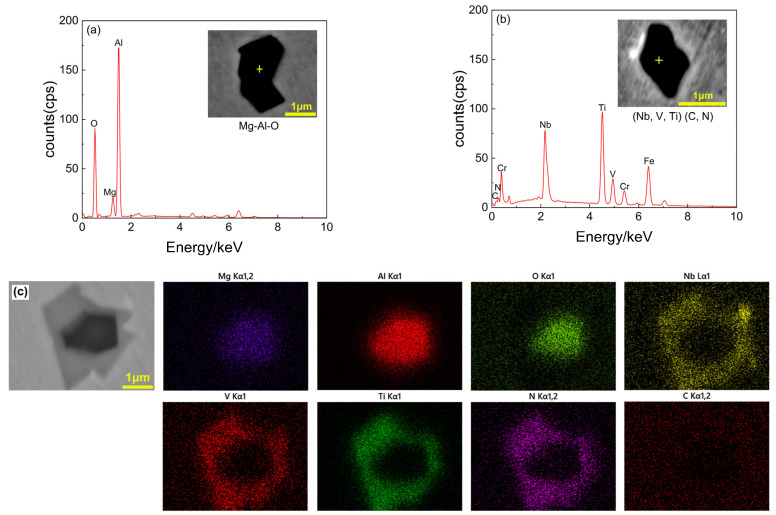
SEM morphology and EDS analysis of typical inclusions in A0 steel: (**a**) Mg-Al-O inclusion; (**b**) (Nb, V, Ti)(C, N) inclusion; (**c**) Mg-Al-O+(Nb, V, Ti)(C, N) composite inclusion.

Figure 1 shows the SEM backscattered electron images and EDS analysis results of typical inclusions in the A0 steel without rare earth addition. Three types of typical inclusions were predominantly observed in the A0 steel: Mg-Al-O system oxides (composed of MgO, Al_2_O_3_ or MgO·Al_2_O_3_), (Nb, V, Ti)(C, N) system carbonitrides (hereinafter referred to as MX phase), and composite inclusions formed by carbonitrides encapsulating oxides, corresponding to Figure 1a–c, respectively. The size of oxides and carbonitrides is small, between 1 and 2 μm, and the size of composite inclusions is large, which is composed of core Mg-Al-O oxides and external carbonitrides, and the size can reach more than 4 μm. Several inclusions have obvious edges and corners, and the existence of such inclusions will also affect the continuity of the material structure, which easily causes the product to crack during rolling or service [19].

**Figure 2:**
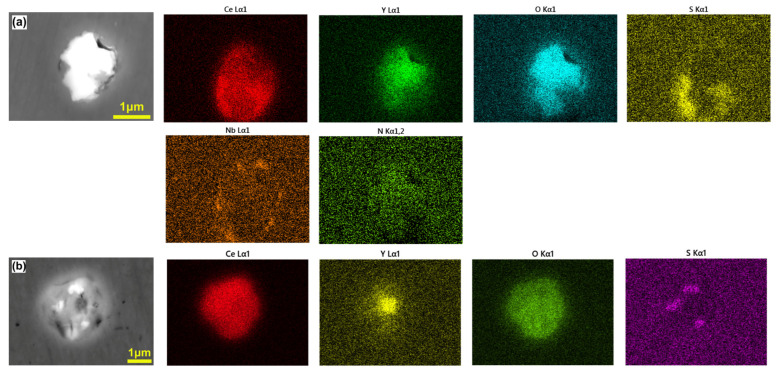
SEM morphology and EDS analysis of typical inclusions in B0 steel: (**a**) Ce-Y-O-S+Nb(C, N) composite inclusions; (**b**) Ce-Y-O-S inclusion.

The backscattered images of typical inclusions and the scanning results of element distribution in B0 steel with 0.015 wt.% Ce + 0.012 wt.% Y are shown in Figure 2.

**Figure 3:**
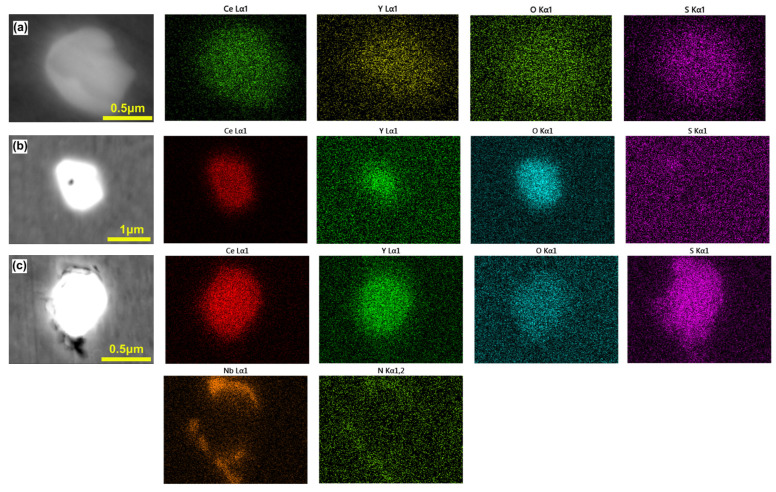
SEM morphology and EDS analysis of typical inclusions in C0 steel: (**a**) Ce-Y-O-S inclusion; (**b**) Ce-Y-O inclusion; (**c**) Ce-Y-O-S+Nb(C, N) composite inclusion.

The main body of the inclusions in Figure 2a is Ce-Y-O rare earth oxide (composed of rare earth oxide), and a small amount of Ce_x_S_y_ sulfide and Nb(C, N) exist outside the inclusions. The main body of the inclusions in Figure 2b is Ce-Y-O rare earth oxide, Y gathers in the center, and there is a small amount of rare earth sulfide in the inner layer. Figure 3 is the backscattered image of typical inclusions and the scanning results of the element distribution surface of C0 steel with 0.022 wt.% Ce + 0.042 wt.% Y.

The inclusions are mainly Ce-Y-O-S (composed of rare earth oxide, sulfide and oxysulfide) and Ce-Y-O inclusions. It is noteworthy that fine and dispersed Nb (C, N) particles are still attached to the outer edges of some inclusions, as shown in Figure 3c. After adding rare earth Ce and Y to the steel, the inclusions in the steel become dispersed spherical inclusions, and the size of the inclusions is significantly reduced, mainly concentrated at about 1 μm, and no single large primary MX phase is found. Studies have shown that the elastic modulus of rare earth inclusions is close to the elastic modulus of the steel matrix, which has little effect on the continuity of the microstructure after forging deformation, which is beneficial to the related properties of the product [9].

### 3.2. Effect of Rare Earth on the Type, Size, and Number Density of Inclusions

**Figure 4:**
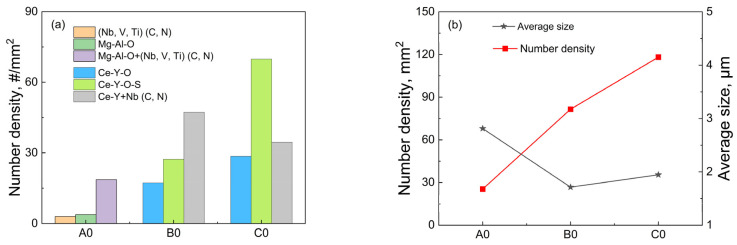
The number and average size, as well as number density and area density of inclusions in T91 steel with different rare earth contents: (**a**) number; (**b**) average size and number density.

Figure 4 shows the statistical results of the composition, quantity, and size of inclusions in the sample analyzed by the ASPEX inclusion automatic analysis software.

Since the statistical lower limit of ASPEX inclusions is set to 0.5 μm, some small-sized inclusions and secondary precipitates are not included in the statistics. It can be seen from Figure 4a that the A0 steel is mainly composed of Mg-Al-O+MX composite inclusions, and the proportion is as high as 73%. With the increase in rare earth content, all Mg-Al-O inclusions disappear, and the number of Ce-Y-O-S inclusions in C0 steel is significantly higher than that in B0 steel, while the number of inclusions containing MX phase decreases, and the proportion of Ce-Y-O oxide inclusions is basically unchanged. Figure 4b shows the statistical results of the number and size of inclusions in different steels. Compared with A0 steel without rare earth, the size of inclusions in B0 and C0 steel decreased significantly from 2.8 μm to 1.7 μm and 1.9 μm, which increased slightly with the increase in rare earth content but remained basically unchanged. The number density of inclusions increases with the increase in rare earth content, from 25/mm^2^ without rare earth to 82/mm^2^ and 118/mm^2^. Combined with Table 1, it can be seen that rare earth combines with O and S to form inclusions in T91 steel. While refining inclusions, some inclusions float and are removed, resulting in a decrease in O and S content in steel, which plays a strong role in purifying molten steel [20].

**Figure 5:**
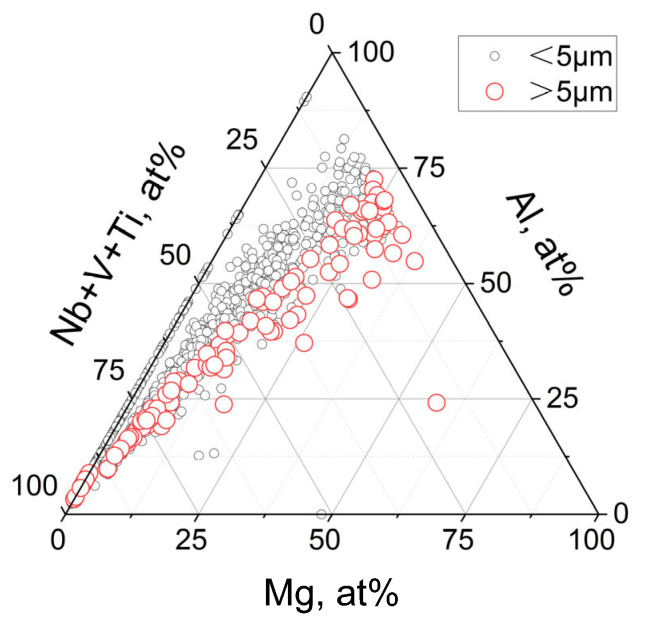
Atomic percentage of Mg-Al-O+(Nb, Ti, V)(C, N) composite inclusions in A0 steel.

Figure 5 shows the statistical results of the atomic percentage of each element in the composite inclusions in A0 steel.

With the increase in Mg content, the size of inclusions gradually increases, and a large number of inclusions with a size of more than 5 μm appear. The reason may be related to the large size oxide inclusions formed by Ni-Mg precipitation deoxidation in the smelting process. During the solidification process, the MX precipitate phase is formed on the surface of the oxide, resulting in a further increase in the size of the inclusions.

**Figure 6:**
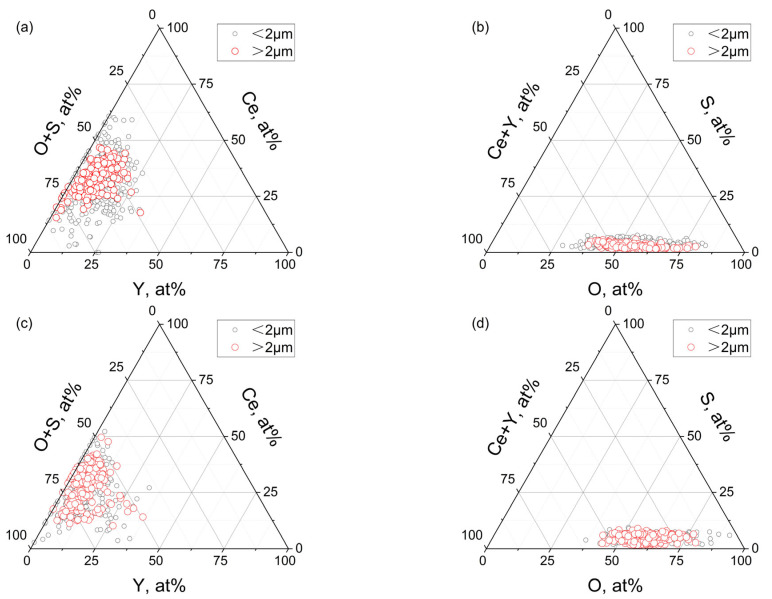
Atomic percentage of Ce-Y-O-S inclusions: (**a**,**b**) B0 steel; (**c**,**d**) C0 steel.

Figure 6 shows the statistical results of the atomic percentage of each element in Ce-Y-O-S inclusions of B0 and C0 steels.

It can be seen from the figure that although the Y/Ce ratio in C0 steel reaches 1.9, which is greatly improved compared with B0 steel (Y/Ce ratio = 0.8), the change in rare earth content has little effect on the distribution of inclusions. The proportion of Ce atoms is between 25 and 50%, the proportion of Y atoms is between 0 and 25%, and the proportion of S atoms is less than 10%. Considering that the sulfur-containing phases formed by rare earth in steel are mainly RE_2_O_2_S, RE_3_S_4_, RE_2_S_3,_ and RES, in which the proportion of S atoms is 20%, 57%, 60%, and 50%, respectively, which are significantly higher than 10%. Therefore, it can be considered that the Ce-Y-O-S inclusions in steel are composed of oxides, sulfides, or sulfur oxides.

### 3.3. Thermodynamic Analysis of Inclusion Formation in T91 Steel

**Figure 7:**
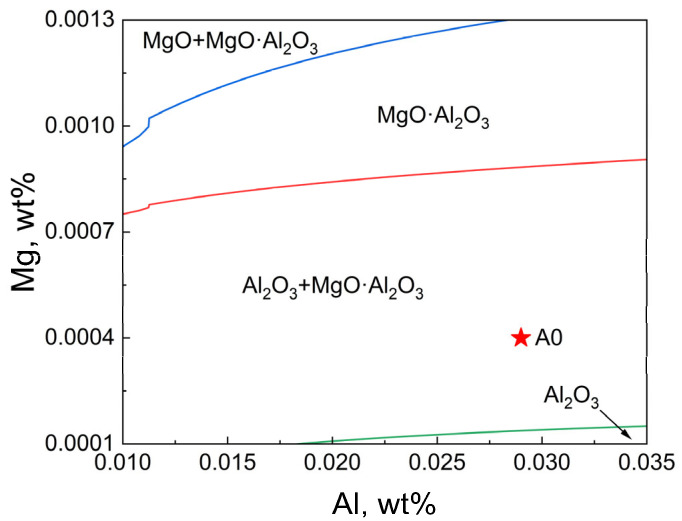
Phase diagram of Mg-Al-O inclusions in T91 steel at 1600 °C, the red asterisk is the composition of A0 steel.

The phase diagram module of FactSage 8.3 thermodynamic software was used to calculate the stable phase diagram of inclusions in molten steel at 1600 °C. The pure material database (FactPS), oxide database (FToxid), steel-based database (FSsteel), and self-built thermodynamic database containing Ce, O, and S were selected. The calculation results without rare earth addition are shown in Figure 7.

Only when the Mg content is less than 0.0001 wt.%, the Al_2_O_3_ inclusions in the molten steel exist stably, while the A0 steel is located in the Al_2_O_3_ + MgO·Al_2_O_3_ inclusion region in the phase diagram, which is consistent with the experimental observation results.

**Figure 8:**
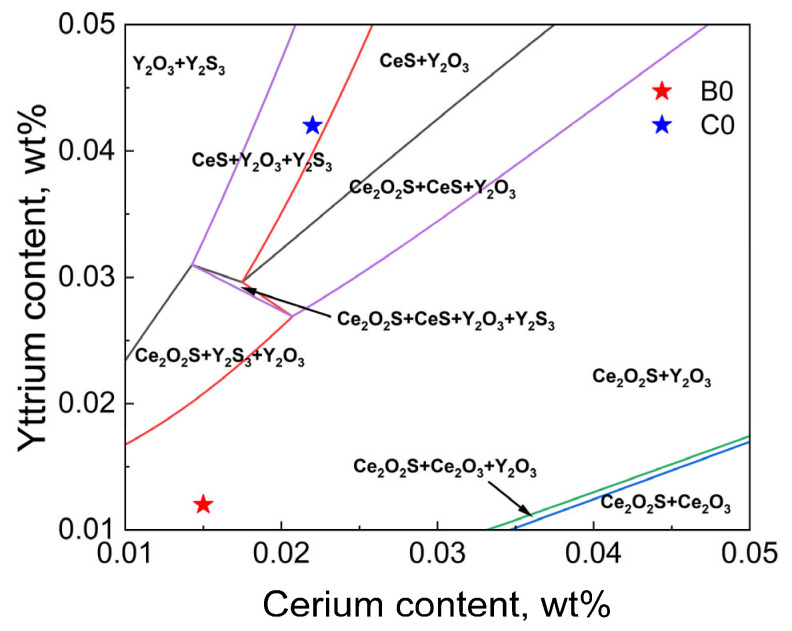
Stable phase diagram of Ce-Y-O-S inclusions in T91 steel at 1600 °C.

The calculation results of the phase diagram of T91 steel after adding rare earth are shown in Figure 8.

When the Y content in the molten steel is in different ranges, the increase in Ce content can make the rare earth inclusions Y_2_S_3_, Y_2_O_3,_ and CeS change to Ce_2_O_2_S and Ce_2_O_3_. When the Y content in the liquid steel is in the range of 0.01 wt.% ~ 0.015 wt.%, the increase in Ce content can change the rare earth inclusion Y_2_O_3_ to Ce_2_O_3_. When the Y content in the liquid steel is in the range of 0.015 wt.% ~ 0.05 wt.%, the increase in Ce content can make Y_2_S_3_ transform to CeS and CeS transform to Ce_2_O_2_S. At the Y content point of B0 steel, Y_2_O_3_→Ce_2_O_3_ transition occurs when the Ce content increases to 0.035 wt.%. At the Y content point of C0 steel, with the increase in Ce content, Y_2_S_3_→CeS→Ce_2_O_2_S transition will occur in turn. According to the electron microscope observation and thermodynamic calculation results, the types of inclusions that may precipitate in B0 and C0 steels are the same, and the types that are easier to precipitate are Ce_2_O_3_, Ce_2_O_2_S, Y_2_O_3_, Y_2_S_3,_ and CeS. Due to the different contents of kinetics, local concentration, alloying stability, and solidification process [9], there may be different contents of Ce-Y-O and Ce-Y-O-S composite inclusions in steel.

Combined with the above typical inclusion observation results and FaceSage thermodynamic calculation results, the possible reactions and corresponding standard Gibbs free energy of Ce and Y after adding molten steel are listed in Table 2.

Table 3 lists the element interaction coefficient of T91 steel at 1873 K.

Since the three samples in this study underwent a long homogenization process before forging, the inclusions were transformed. According to the study of Ahmad et al. [23] and Zhang et al. [17], one method for the transformation of rare earth inclusions during the heating process at 1473 K is the internal transformation caused by glassy crystallization. This transformation strongly depends on the type of rare earth ions. Y has a smaller ion radius than Ce, which means a higher charge density, which leads to an increase in the bond strength in the glass network, thereby increasing the temperature of glass transition and crystallization so that Y is more likely to aggregate in the inner layer, which corresponds to the inclusion in Figure 2b. Another method is that the interface reaction between inclusions and steel matrix leads to the transformation of inclusions. With the decrease in temperature, the solubility of rare earth in steel matrix decreases [24,25]. Since the inclusions in molten steel are mainly Y_2_O_3_ and Y_2_S_3_, the diffusion reaction of Ce to inclusions in the matrix during homogenization should be considered. According to the actual electron microscope observation results, it is speculated that the possible reactions during the heating process are shown in Equations (1)–(5).

(1) $${\left[\mathrm{Ce}\right]}_{\left(\mathrm{solid}\;\mathrm{steel}\right)}+\frac{1}{3}{Y_{2}S_{3}}_{\left(\mathrm{inclusion}\right)}{=\mathrm{CeS}}_{\left(\mathrm{inclusion}\right)}+\frac{2}{3}{\left[Y\right]}_{\left(\mathrm{solid}\;\mathrm{steel}\right)}\;\;\;\;\;\;\;\;\;\;\;\;\;\;\;\;\;\;\;\;\;\;\;\;\Delta G_{10}^{\theta }=-31767-26.62T$$

(2) $${\left[\mathrm{Ce}\right]}_{\left(\mathrm{solid}\;\mathrm{steel}\right)}+\frac{1}{2}{Y_{2}S_{3}}_{\left(\mathrm{inclusion}\right)}=\frac{1}{2}{{\mathrm{Ce}}_{2}S_{3}}_{\left(\mathrm{inclusion}\right)}{+[Y]}_{\left(\mathrm{solid}\;\mathrm{steel}\right)}\;\;\;\;\;\;\;\;\;\;\;\;\;\;\;\;\;\;\;\;\;\Delta G_{11}^{\theta }=49080-56.64T$$

(3) $${\left[\mathrm{Ce}\right]}_{\left(\mathrm{solid}\;\mathrm{steel}\right)}+\frac{4}{9}{Y_{2}S_{3}}_{\left(\mathrm{inclusion}\right)}=\frac{1}{3}{{\mathrm{Ce}}_{3}S_{4}}_{\left(\mathrm{inclusion}\right)}+\frac{8}{9}{\left[Y\right]}_{\left(\mathrm{solid}\;\mathrm{steel}\right)}\;\;\;\;\;\;\;\;\;\;\;\;\;\Delta G_{12}^{\theta }=22774-49.7T$$

(4) $${\left[\mathrm{Ce}\right]}_{\left(\mathrm{solid}\;\mathrm{steel}\right)}+\frac{1}{6}{Y_{2}S_{3}}_{\left(\mathrm{inclusion}\right)}+\frac{1}{3}{Y_{2}O_{3}}_{\left(\mathrm{inclusion}\right)}=\frac{1}{2}{{\mathrm{Ce}}_{2}O_{2}S}_{\left(\mathrm{inclusion}\right)}{+[Y]}_{\left(\mathrm{solid}\;\mathrm{steel}\right)}\;\;\;\;\;\;\;\;\;\;\;\Delta G_{13}^{\theta }=117000-127.33T$$

(5) $${\left[\mathrm{Ce}\right]}_{\left(\mathrm{solid}\;\mathrm{steel}\right)}+\frac{1}{2}{Y_{2}O_{3}}_{\left(\mathrm{inclusion}\right)}=\frac{1}{2}{{\mathrm{Ce}}_{2}O_{3}}_{\left(\mathrm{inclusion}\right)}{+[Y]}_{\left(\mathrm{solid}\;\mathrm{steel}\right)}\;\;\;\;\;\;\;\;\;\;\;\;\;\;\;\;\;\;\;\;\Delta G_{14}^{\theta }=181920-149.26T$$

**Figure 9:**
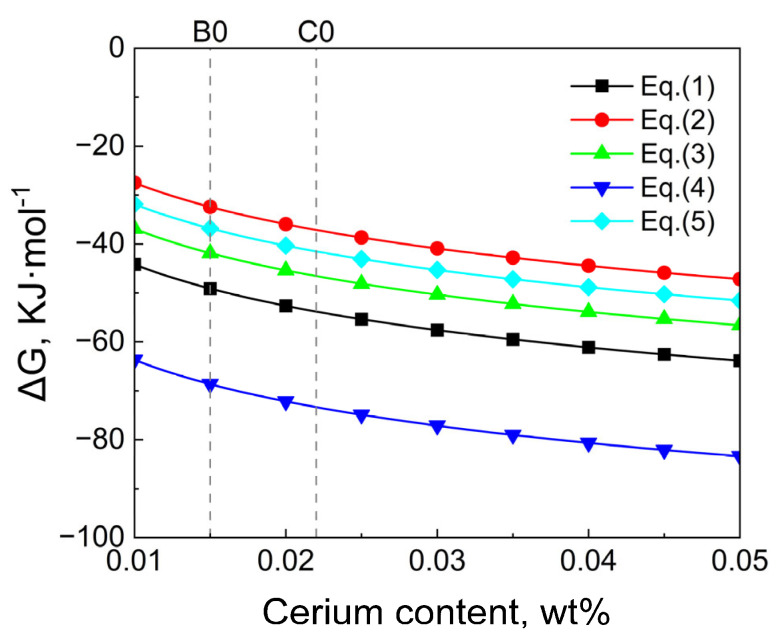
Gibbs free energy change of the transformation of rare earth inclusions under the heating condition at 1200 °C.

Due to the lack of thermodynamic data of the Y-Ce-Fe-O system at 1473 K, it is assumed that the relevant data in the molten iron are applicable to this temperature [17], and the activity correlation coefficient and standard Gibbs free energy at 1873 K are still used for calculation. According to the above reaction formula and the activity coefficients of each element in molten steel in Table 3, combined with the Wagner model (Equations (6)–(8)), the Gibbs free energy of different reactions at 1473 K is calculated as shown in Figure 9, where the S and O contents are 0.001 wt.%, and the Y content is 0.015 wt.%.

(6) $$a_{i}=f_{i}[\%i]$$

(7) $$\mathrm{lg}f_{i}=\sum_{i=1}^{n}e_{i}^{j}[\%j]$$

(8) $$\Delta G=\Delta G^{\theta }+\mathrm{RT}\ln Q$$

where Δ*G* is the Gibbs free energy. Δ*G*^θ^ is the Gibbs free energy at the same temperature and standard pressure. R is the gas universal constant, 8.314 J/(K·mol). T is the reaction temperature, K. Q is activity entropy, J/(mol·K). *f*_i_ is the activity coefficient of element *i*. a*_i_* is the activity of element *i*, [%*i*] and [%*j*] are the mass fractions of *i* and *j*, respectively. $e_{i}^{j}$ is the activity interaction coefficient of *i* and *j*.

The calculated results are all negative, indicating that from a thermodynamic point of view, all five reactions can occur after the addition of Ce, indicating that during the heat preservation process at 1473 K, the diffusion reaction of Ce from the matrix to the inclusions can occur between the rare earth inclusions and the steel matrix. The reaction (1) is significantly greater than (2) and (3), indicating that T91 steel is more likely to generate CeS during the 24-h homogenization process than Ce_2_S_3_ and Ce_3_S_4_. In addition, the Gibbs free energy of the conversion reaction to generate Ce_2_O_2_S is the most negative, less than 60 KJ·mol^−1^, indicating that the Y_2_O_3_ + Y_2_S_3_ → Ce_2_O_2_S reaction is most likely to occur. Therefore, more such inclusions can be observed in the sample. After the diffusion reaction occurs, the inclusion presents the element distribution of Ce-wrapped Y. According to the results of ASPEX inclusion automatic scanning composition distribution, the Ce content of inclusions in B0 and C0 steels is significantly higher than that of Y content. This further confirmed that the diffusion reaction of Ce to inclusions in the matrix easily occurs during long-term heating, resulting in the transformation of rare earth inclusions Y_2_S_3_, Y_2_O_3,_ and CeS to Ce_2_O_2_S and Ce_2_O_3_ to a certain extent.

Studies have shown that the diffusion of dissolved rare earth elements in molten steel is a limiting factor affecting the type and growth of modified inclusions [12]. In addition, the growth rate of inclusions is related to the concentration of elements around the inclusions during the diffusion process from the reactants to the core of the inclusions [12], as shown in Equation (9):

(9) $$\frac{R^{2}}{t}=D(\frac{c_{0}-c_{e}}{2c_{p}})$$

where *R* is the inclusion radius, m. *t* is time, s. *D* is the diffusion coefficient of the reaction element, m^2^/s. *c*_0_ is the initial concentration of reaction elements in molten steel, kg/m^3^. *c_e_* is the concentration of reaction elements in molten steel at equilibrium, kg/m^3^. *c_p_* is the concentration of elements in the inclusions. On the one hand, the increase in Ce and Y addition will increase *c*_0_, on the other hand, the extension of holding time will lead to the decrease in *c_e_*, both of which are factors that cause the growth rate and size of inclusions to increase. Therefore, excessive addition of rare earth should be avoided in industrial production to prevent the formation of large-size inclusions [12]. Yin et al. [26] proposed that the “maximum acting distance” can be used to characterize the agglomeration tendency of various inclusions. Wang et al. [27] compared the agglomeration attraction of four different inclusions. The attractive force of Al_2_O_3_ inclusions is the strongest, and it is easiest to form large-sized clusters. Although the attractive force of Ce-Al-O and Ce_2_O_3_ inclusions is weaker than that of Al_2_O_3_, they can still agglomerate to form clusters, and the attraction between Ce-O-S inclusions is the weakest, that is, they are more difficult to agglomerate than Al_2_O_3_, Ce-Al-O and Ce_2_O_3_ inclusions. From the above statistical results of inclusions, it can be found that the size of inclusions decreases significantly from 2.8 μm to 1.7 μm after adding rare earth in T91 steel, which is mainly due to the weak agglomeration attraction of rare earth inclusions. When the rare earth content increases from 0.015 wt.% Ce + 0.012.wt.% Y to 0.022 wt.% Ce + 0.042 wt.% Y, the average size of inclusions only increases by 0.2 μm. On the one hand, the increase in Ce and Y addition will increase the *c*_0_ in Equation (5), resulting in an increase in the growth rate and size of inclusions. On the other hand, due to the formation of more Ce-Y-O-S inclusions with low agglomeration attraction, the size of inclusions is reduced. Due to the combined effect of the two mechanisms, although the rare earth content in the steel has more than doubled, the size of the inclusions has not increased much.

After adding a certain amount of rare earth Ce and Y to T91 steel, the Mg-Al-O inclusions in the steel can be modified into high melting point rare earth oxides, rare earth sulfides, and rare earth oxysulfide inclusions. The size of the inclusions is reduced compared with that without rare earth elements, indicating that rare earth elements have the effect of refining inclusions. Since these small particles with high melting points are not easy to fuse, the reduction in the original size of the inclusions directly leads to a significant decrease in their binding force (van der Waals force). At the same time, the decrease in reaction element concentration, nucleation number, and inclusion size also directly reduces the collision constant and ultimately reduces the final size of the inclusion [9]. The inclusions modified by rare earth not only have beneficial changes in size, quantity, and morphology, but also their mechanical properties are more in line with the steel matrix than traditional inclusions. Li et al. [8] obtained the elastic modulus, Young’s modulus, shear modulus, and hardness of these rare earth inclusions by first-principles calculations, which are much lower than those of traditional Al inclusions. This kind of small size and spherical rare earth inclusions has a similar linear expansion coefficient with the steel matrix, and it is difficult to deform during the plastic deformation of the steel. Therefore, it is not easy to produce stress concentration around such rare earth inclusions during the hot processing of steel, which effectively reduces the crack formation ability and propagation ability at inclusions and improves the strength and toughness of steel.

### 3.4. Analysis of the Influence Mechanism of Inclusions on Precipitated Phase

**Figure 10:**
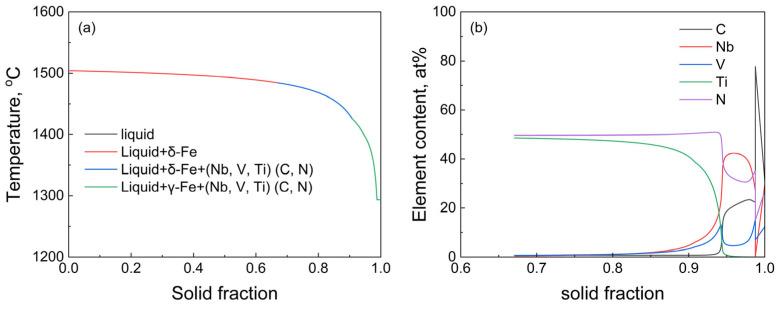
Calculation of nonequilibrium solidification with the Scheil–Gulliver model in Thermo-Calc 2023a software: (**a**) solidification curve of T91 steel ingot; (**b**) variation of (Nb, V, Ti) (C, N) phase-forming elements.

The formation and transformation of precipitates during non-equilibrium solidification of T91 steel were predicted by the Scheil-Gulliver model included in Thermo-Calc software 2023a (TCFE10 database). The results are shown in Figure 10.

The solidus and liquidus temperatures are 1504 °C and 1293 °C, respectively. When the solidification temperature and solid fraction reach 1484 °C and 0.67, respectively, (Nb, V, Ti)(C, N) phase will precipitate in the molten steel. When the solidification temperature and solid fraction reach 1425 °C and 0.91, respectively, peritectic transformation occurs. In addition, it can be seen from Figure 10b that TiN is mainly precipitated in the early stage of solidification, and (Nb, V) (C, N) is precipitated in the late stage of solidification.

According to the thermodynamic calculation of FactSage, T91 steel can form solid oxide, sulfide, and sulfur oxide inclusions above the liquidus temperature and then gradually precipitate carbonitride during solidification. Therefore, these inclusions are in line with the prerequisites for playing the role of heterogeneous nucleation core. The misfit is defined as the relative difference in the atomic spacing between adjacent two-phase interfaces that determines the size of the elastic strain energy on the coherent crystal plane. The two-dimensional mismatch can be used to characterize the matching relationship between different lattice types of materials in molten steel, between ferrite and austenite, and between different types of inclusions. Bramfitt [28] established a mismatch model between different types of base phases and nucleation phases, which can be used to calculate the matching degree and mutual binding ability of different types of inclusions in molten steel. The calculation formula is as follows:

(10) $$\delta_{{\left(\mathrm{hkl}\right)}_{n}}^{{\left(\mathrm{hkl}\right)}_{s}}=\overset{3}{\underset{i=1}{\sum }}\frac{\frac{\left|\left({d[\mathrm{uvw}]}_{s}^{i}\cos\theta \right){-d[\mathrm{uvw}]}_{n}^{i}\right|}{{d[\mathrm{uvw}]}_{n}^{i}}}{3}\times 100\%$$

In the formula, (hkl)_s_ is a low-index crystal plane of the base phase. [uvw]_s_ is a low-index direction on the crystal plane (hkl)_s_. (hkl)_n_ is a low-index crystal plane of the nucleation phase. [uvw]_n_ is a low-index direction on the crystal plane (hkl)_n_. d[uvw]_s_ is the atomic spacing along the [uvw]_s_ direction. d[uvw]_n_ is the atomic spacing along the [uvw]_n_ direction. θ is the angle between [uvw]_s_ and [uvw]_n_.

In order to analyze the possibility of heterogeneous nucleation of carbonitrides induced by different types of inclusions during solidification of molten steel, it is necessary to master the crystallographic information of inclusions and carbonitrides. The crystallographic parameters of inclusions and carbonitrides were obtained by referring to the ICSD inorganic crystal structure database, as shown in Table 4. It should be pointed out that for simplicity, since the crystal structures of NbN, VN, TiN, NbC, VC, and TiC are consistent with the spatial lattice group and their lattice constants are similar, NbN is selected as a representative to calculate the mismatch between carbonitrides and inclusions. Because the structure of Y_2_S_3_ is complex and the content is low, it is not calculated here. The lattice constants of each phase used in the calculation are the lattice constants at room temperature, and the effect of the thermal expansion coefficient on the lattice constants is ignored.

According to the two-dimensional mismatch model proposed by Bramfitt [28], when the lattice mismatch is less than 6%, the heterogeneous nucleation effect is the best; when the lattice mismatch is between 6% and 12%, the heterogeneous nucleation effect is moderate; when the lattice mismatch is greater than 12%, heterogeneous nucleation is difficult to occur, and the angle between the crystal orientations should not be obtuse. The calculation results of the mismatch degree are shown in Table 5. The lattice mismatch between (100) NbN and (0001) Al_2_O_3_ in the low index plane of A0 steel without rare earth is the lowest and only 6.2, indicating that NbN has the best heterogeneous nucleation effect on Al_2_O_3_. The (Nb, V, Ti)(C, N) with Mg-Al-O inclusion as the core growing in the outer layer is precipitated and grown up when the actual concentration product is greater than the equilibrium concentration product during the solidification of molten steel. The lattice mismatch between the low index plane (0001) Ce_2_O_3_ and (100) NbN is 9.3, and the nucleation effect is moderate. The lattice mismatch between Ce_2_O_2_S, Y_2_O_3,_ and NbN is 13.3 and 13.5, respectively, so NbN is relatively difficult to nucleate on Ce_2_O_2_S and Y_2_O_3_. The mismatch between NbN and CeS is the largest, which is 18.9, so NbN can hardly nucleate on CeS. Therefore, it is difficult for NbN to heterogeneously nucleate on rare earth inclusions during solidification, and only a small amount of NbN precipitates on the surface, and no large primary carbonitride precipitates in the experimental observation.

## 4. Conclusions

In this paper, the modification effect of rare earth elements Ce and Y on inclusions in T91 heat-resistant steel was studied, and the evolution mechanism of inclusions in molten steel, the heating process, and the solidification process was analyzed by thermodynamic and kinetic simulation. The following conclusions are drawn:

1. The addition of rare earth Ce and Y makes the inclusions in T91 heat-resistant steel modified from angular Mg-Al-O oxides, (Nb, V, Ti)(C, N) carbonitrides and Mg-Al-O+(Nb, V, Ti)(C, N) composite inclusions to spherical Ce-Y-O, Ce-Y-O-S inclusions, and Ce-Y+Nb(C, N) composite inclusions. Compared with the sample without rare earth elements, after adding 0.015 wt.% Ce + 0.012 wt.% Y and 0.022 wt.% Ce + 0.042 wt.% Y, the number density of inclusions in T91 heat-resistant steel increased from 25/mm^2^ to 82/mm^2^ and 118/mm^2^, while the average size decreased significantly from 2.8 μm to 1.7 μm and 1.9 μm. It can be seen that due to the combined effect of the diffusion mechanism and the agglomeration mechanism, the further increase in rare earth content in a certain range will make the size of rare earth inclusions have an increasing trend.
2. Thermodynamic calculations show that after the T91 heat-resistant steel with Ce and Y added undergoes homogenization at 1200 °C for 24 h and forging, the internal transformation of inclusions causes Y to accumulate in the inner layer. Meanwhile, the diffusion of Ce into inclusions in the matrix occurs Y_2_S_3_→CeS and Y_2_O_3_ + Y_2_S_3_→Ce_2_O_2_S. Eventually, the inclusion layer containing Ce is formed on the surface of inclusions. Therefore, it can be inferred that the increase in Ce content in steel makes the above reaction more likely to occur.
3. During the solidification process of T91 heat-resistant steel, primary (Nb, V, Ti)(C, N) phases precipitate. However, the primary precipitation phase has not been detected in the steel after adding rare earths, and with the increase in the content of rare earths, the amount of Nb-containing composite phases on the surface of inclusions decreases. Combined with the calculation of the lattice mismatch, only Ce_2_O_3_ has a moderate heterogeneous nucleation effect with (Nb, V, Ti)(C, N). Therefore, appropriately increasing the Y/Ce ratio can reduce the formation of Ce_2_O_3_ and further avoid the formation of the primary precipitation phase (Nb, V, Ti)(C, N), which helps to improve the dispersion strengthening effect of the nanoscale secondary precipitation phase MX. Therefore, it is necessary to study the effect of different Y/Ce ratios on the size, number, and distribution of nano-scale secondary precipitated phase MX, so revealing the mechanism of rare earth elements in enhancing creep strength and steam oxidation resistance, thereby improving the service performance of T91 steel.

## Figures and Tables

**Table 1:** Chemical compositions of T91 steel (wt.%).

Steel	C	Si	V	Cr	Mo	Nb	Mn	Al	Ti	N	O	S	Ce	Y
A0	0.10	0.49	0.22	9.36	1.02	0.073	0.50	0.029	0.0006	0.047	0.0022	0.001	0	0
B0	0.11	0.49	0.20	9.05	0.97	0.070	0.46	0.0044	0.0015	0.039	0.0012	0.0012	0.015	0.012
C0	0.10	0.54	0.21	9.24	1.05	0.065	0.46	0.0045	0.0010	0.066	0.0006	0.0014	0.022	0.042

**Table 2:** Possible reactions in the molten steel, corresponding standard Gibbs free energy, and Gibbs free energy at 1873K [9,21,22].

Formula of Reactions	${\Delta G}^{\theta },\;{\mathrm{Jmol}}^{-1}$(1873 K)
${[\mathrm{Ce}]+3/2[O]=1/2\mathrm{Ce}}_{2}O_{3}\left(s\right)$	${\Delta G}_{1}^{\theta }=-714380+179.74T$
[Ce]+[S]=CeS(s)	${\Delta G}_{2}^{\theta }=-422100+120.38T$
${[\mathrm{Ce}]+3/2[S]=1/2\mathrm{Ce}}_{2}S_{3}$	${\Delta G}_{3}^{\theta }=-536420+163.86T$
${[\mathrm{Ce}]+4/3[S]=1/3\mathrm{Ce}}_{3}S_{4}\left(s\right)$	${\Delta G}_{4}^{\theta }=-497670+146.30T$
${[\mathrm{Ce}]+1/2[O]+[S]=1/2\mathrm{Ce}}_{2}O_{2}S(s)$	${\Delta G}_{5}^{\theta }=-675700+165.50T$
${[Y]+3/2[O]=1/2Y}_{2}O_{3}\left(s\right)$	${\Delta G}_{6}^{\theta }=-896300+329T$
[Y]+[S]=YS(s)	${\Delta G}_{7}^{\theta }=-321080+91.0T$
${[Y]+3/2[S]=1/2Y}_{2}S_{3}\left(s\right)$	${\Delta G}_{8}^{\theta }=-585500+220.5T$
${[Y]+[O]+1/2[S]=1/2Y}_{2}O_{2}S(s)$	${\Delta G}_{9}^{\theta }=-760500+268.0T$

**Table 3:** The first interaction coefficients in this study.

(i,j)	C	N	O	Si	Mn	S	Al	Ce	Y
O	−0.45	0.057	−0.2	−0.131	−0.021	−0.133	−3.9	−0.57	−16.3
Ce	0.397	−6.612	−5.03		0.13	−10.34	−2.67	−0.008	
S	0.11	0.01	−0.27	0.063	−0.026	−0.028	0.035	−2.36	−0.55
Al	0.091	−0.058	−6.6	0.0056	0.035	0.03	0.045	−0.5114	
Y	−0.22	−3.55	−90.7			−7.34			−0.006

**Table 4:** Calculation parameters of mismatch degree.

Phase	Crystal Type	Lattice Parameters (10^−10^ m)
BCC	cubic	a = b = c = 2.867
Al_2_O_3_	hexagonal	a = b = 4.759, c = 12.99
NbN	cubic	a = b = c = 4.442
TiN	cubic	a = b = c = 4.235
Ce_2_O_3_	hexagonal	a = b = 3.891, c = 6.059
Ce_2_O_2_S	hexagonal	a = b = 4.002, c = 6.888
Y_2_O_3_	cubic	a = b = c = 10.607
CeS	cubic	a = b = c = 5.776

**Table 5:** Calculation results of mismatch degree.

Inclusion Type	Inclusion//NbN	[uvw]_s_	[uvw]_n_	d[uvw]_s_	d[uvw]_n_	θ	δ
Al_2_O_3_	(0001)//(100)	$\left[\bar{1}2\bar{1}0\right]$	[100]	4.759	4.442	0	6.2
		$\left[\bar{2}110\right]$	[102]	4.759	4.966	3.43	
		$\left[\bar{1}010\right]$	[001]	8.243	8.884	0	
Ce_2_O_3_	(0001)//(110)	$\left[\bar{1}2\bar{1}0\right]$	[001]	3.891	4.442	0	9.3
		$\left[\bar{2}110\right]$	$\left[1\bar{1}1\right]$	3.891	3.847	5.26	
		$\left[\bar{2}110\right]$	$\left[2\bar{2}1\right]$	7.782	6.663	10.53	
Ce_2_O_2_S	(0001)//(110)	$\left[\bar{1}2\bar{1}0\right]$	[001]	4.002	4.442	0	13.3
		$\left[\bar{2}110\right]$	$\left[1\bar{1}1\right]$	4.002	3.847	5.26	
		$\left[\bar{1}010\right]$	$\left[1\bar{1}0\right]$	6.932	9.423	0	
Y_2_O_3_	(111)//(110)	$\left[1\bar{1}0\right]$	$\left[1\bar{1}0\right]$	7.500	3.141	0	13.5
		$\left[1\bar{2}1\right]$	$\left[1\bar{1}1\right]$	12.991	3.847	15	
		$\left[0\bar{1}1\right]$	[001]	7.500	2.221	30	
CeS	(111)//(110)	$\left[1\bar{2}1\right]$	$\left[1\bar{1}1\right]$	7.074	3.847	15	18.9
		$\left[0\bar{1}1\right]$	[001]	4.084	2.221	30	
		$\left[0\bar{1}1\right]$	$\left[1\bar{1}2\right]$	4.084	5.441	5.26	

## Data Availability

The original contributions presented in this study are included in the article. Further inquiries can be directed to the corresponding authors.
